# Co-expression of DKK-1 and Sclerostin in Subchondral Bone of the Proximal Femoral Heads from Osteoarthritic Hips

**DOI:** 10.1007/s00223-017-0246-7

**Published:** 2017-03-09

**Authors:** Allahdad Zarei, Philippa A. Hulley, Afsie Sabokbar, M. Kassim Javaid

**Affiliations:** Botnar Research Centre, Nuffield Department of Orthopaedics, NDORMS, Rheumatology and Musculoskeletal Sciences, University of Oxford, Old Road, Oxford, OX3 7LD UK

**Keywords:** Osteoarthritis, Subchondral bone, DKK-1, SOST, Wnt

## Abstract

**Background:**

Osteoarthritis (OA) is a progressively degenerative joint disease influenced by structural and metabolic factors. There is growing evidence that subchondral bone is involved in both symptomatic and structural progression in OA. The Wnt pathway has been implicated in the progression of OA but the expression and function of the Wnt inhibitors, Dikkopf (DKK-1) and sclerostin (SOST), are unclear.

**Methods:**

We examined the regional distribution of DKK-1 and SOST in subchondral bone of the femoral head using resection specimens following arthroplasty in patients presenting with end-stage OA. Cylindrical cores for immunohistochemistry were taken through midpoint of full thickness cartilage defect, partial cartilage defect, through base of osteophyte and through macroscopically normal cartilage.

**Results:**

Subchondral bone was thickest in cores taken from regions with full cartilage defect and thinnest in cores taken from osteophyte regions. In subchondral bone, expression of both DKK-1 and SOST was observed exclusively in osteocytes. Expression was highest in subchondral bone in cores taken from regions with partial but not full thickness cartilage defects. DKK-1 but not SOST was expressed by chondrocytes in cores with macroscopically normal cartilage.

**Conclusion:**

The current study describes the regional cellular distribution of SOST and DKK-1 in hip OA. Expression was highest in the osteocytes in bone underlying partial thickness cartilage defects. It is however not clear if this is a cause or a consequence of alterations in the overlying cartilage. However, it is suggestive of an active remodeling process which might be targeted by disease-modifying agents.

## Background

There is a growing body of evidence demonstrating changes in the architecture in subchondral bone underlying OA cartilage lesions, which may contribute to the pathogenesis of both structural and symptomatic features of OA [1]. The principal role of the subchondral bone is mechanical with the cortical and trabecular bone compartments continually responding to loads applied to them by remodeling, via the osteocyte network and Wnt signaling [2–4]. The resulting changes in the mechanical properties of the subchondral plate determine, in part, the load exposure of the cartilage at the joint surface leading to a dynamic interplay between loading and bone structure [5].

OA is a disease involving cartilage damage, changes in underlying subchondral bone, osteophyte formation, and inflammation of the joint with unknown factors that initiate these changes [6]. Our current knowledge of the factors that may be involved in the progression of OA includes weight bearing with aging, increased loading from obesity, and previous injuries [7, 8]. The search for other factors that may be involved in cartilage degradation accompanied by alterations in underlying bone has introduced new directions for investigations focused on signaling pathways such as Wnt-frizzled pathways and associated protein regulators. The Wnt-β-catenin pathway is involved during embryogenesis of the joint and skeletal homeostasis during adulthood [9–11]. The Wnt signaling pathway is known to be associated with responses to mechanical degradation in cartilage [12]. Normally in the absence of extracellular signals, cytoplasmic β-catenin is bound to oligomeric complexes that facilitate β-catenin phosphorylation and consequently its proteolytic degradation [13]. In the presence of extracellular ligands unphosphorylated β-catenin accumulates in the cell cytoplasm and then translocates into the nucleus, binding to transcription factors that activate target signals [14].

Regulation of the Wnt pathway involves natural extracellular inhibitors such as DKK-1 and SOST [15, 16]. Mutations that augment the Wnt signaling pathway and stop its interaction with DKK-1 have been shown to be associated with an increase in bone mass density in human adults [17]. Loss of function and mutation of the *SOST* gene is associated with sclerosteosis and Van Buchem disease with a progressive bone growth and high bone mineral density [18].

Differential expression of Wnt proteins and Wnt inhibitors has been shown in OA, and excessive Wnt signaling has also been thought to contribute to cartilage degradation [19, 20]. Blockage of DKK-1 with anti DKK-1 antibody has been shown to increase bone formation in a mouse model of rheumatoid arthritis [21]. Elevated serum levels of DKK-1 in humans has been shown to be associated with reduced risk of OA progression as well as reduce risk of joint space narrowing that consequently leads to cartilage degradation [4, 22]. DKK-1 suppresses chondrocyte hypertrophy, reduces type X collagen expression, but enhances ADAMTS-5 (a disintegrin and metalloproteinase with thrombospondin motifs) and MMP-13 (matrix metalloproteinase 13) expression [23, 24]. However, systemic inhibition of DKK-1 and its overexpression in chondrocytes is shown to diminish development of OA [25, 26].

There is little information on the role of the Wnt antagonist SOST in OA. Reduction in the number of SOST-positive osteocyte cells has been noted to be associated with increased bone density in the femoral neck of hip OA patients [27]. SOST expression is shown to be down-regulated by mechanical loading [28] but can be up-regulated by pro-inflammatory cytokines [29]. Recently SOST expression in mineralized chondrocytes in human growth plate and in articular cartilage biopsies in patients with end-stage OA has been reported [30, 31]. Histologically, human knee chondrocytes and osteocytes in trabecular bone has been shown to express SOST [32]. Furthermore, SOST expression in chondrocytes has been shown to be up-regulated the area of cartilage damage in animal model of OA, and its expression was shown to be decreased in osteocytes residing in subchondral bone associated with bone sclerosis in those animals [32].

To date, there have been no histological studies on the expression of SOST and DKK-1 in human hip OA, although there is extensive evidence of bone remodeling associated with the destructive loss of cartilage. The aims of the current study were firstly to map the cellular distribution of these two Wnt antagonists in relation to the zones of maximal, partial, and no cartilage damage, as well as in the pathological bone remodeling sites of sclerotic bone and osteophyte.

## Materials and Methods

### Immunohistochemistry

**Fig. 1:**
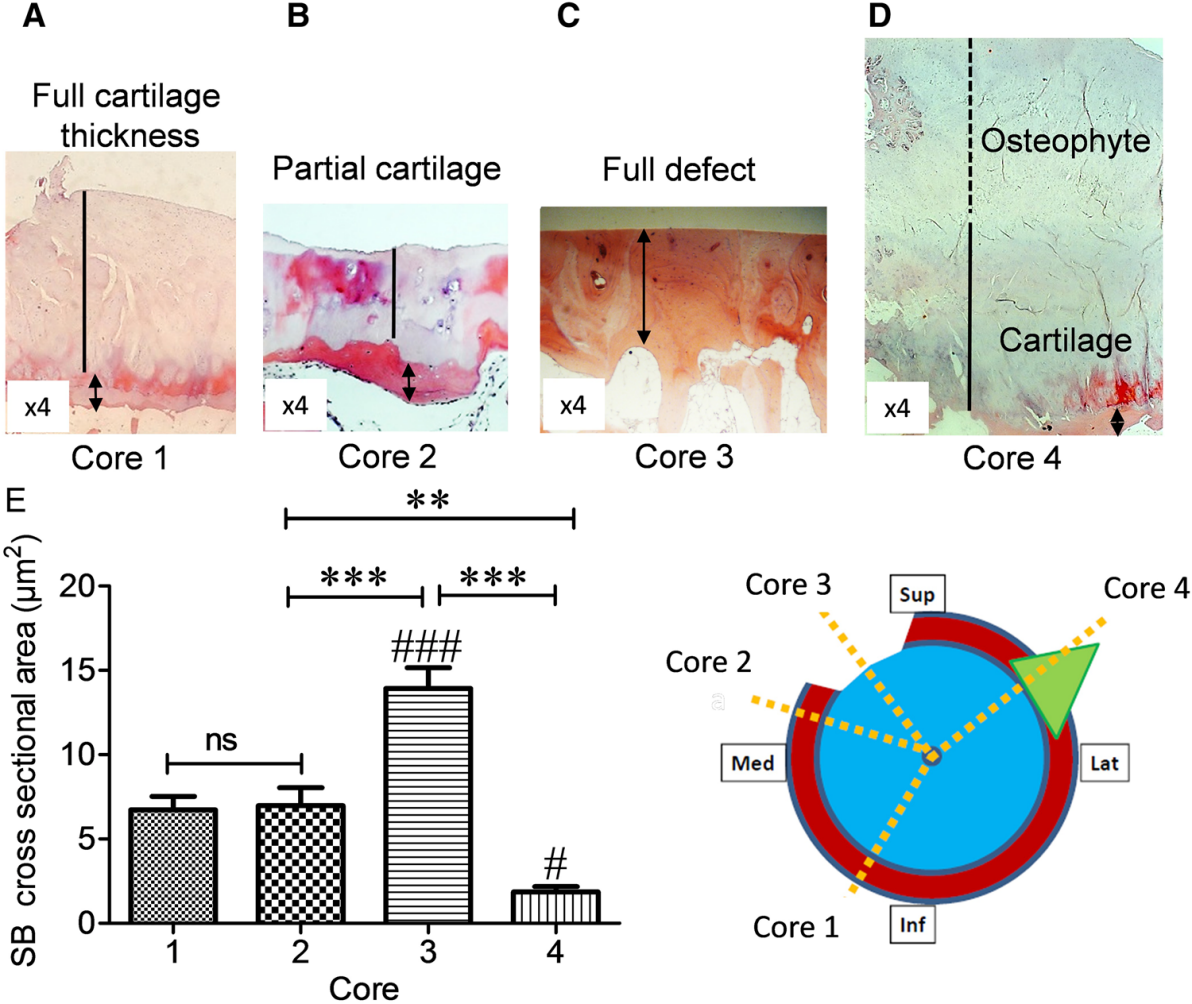
Subchondral bone thickness varies in OA femoral head. Cylindrical cores were taken from 4 OA femoral head biopsies. *Insert cartoon* represents the proposed taken sections of femoral head. *Core 1* macroscopically normal cartilage, *Core 2* partial cartilage defect, *Core 3* full cartilage defect, *Core 4* osteophyte. Representative images of *H&E* staining from **a** macroscopically normal cartilage, **b** partial cartilage defect, **c** full cartilage defect, and **d** osteophyte. **e** Cross-sectional areas of subchondral bone thickness measured by ImageJ software (µm^2^). Data are presented as mean ± SEM (One-way analysis of variance, Tukey’s multiple comparison test) from six slides per each core from four femoral head biopsies. (^#^ *P* < 0.05 and ^###^ *P* < 0.001 for comparison of subchondral bone between full cartilage defect in cores 3 and osteophytes in cores 4 with macroscopically normal cartilage in cores 1, **P < 0.01, and ***P < 0.001 for comparison of core 3 to other cores). Magnifications in **a**–**d** (×4), dashed line osteophyte, solid line cartilage and arrowheads indicate subchondral bone. Not significant (ns), superior (Sup), inferior (Inf), median (Mid), and lateral (Lat)

Human femoral head samples were collected from 4 male patients aged 50–55 year undergoing total hip replacement. Cylindrical perpendicular bone cores (6 × 10 mm^2^ diameter) were taken longitudinally (Fig. 1); through midpoint of full thickness defect, margin of full thickness defect, through base of osteophyte, through macroscopically “normal cartilage”, fixed in 4% paraformaldehyde at 4 °C overnight, decalcified in 0.5 M ethylenediaminetetraacetic acid (Lonza, UK) solution over 6 weeks and embedded in paraffin wax. All cores for histological analysis were taken from the same position on the femoral heads relative to the full thickness defect or osteophyte, and the anatomical locations were confirmed clinically prior to cores being taken. Cores are not in the same plane within each specimen. Paraffin embedded human bone samples were cut longitudinally in serial sections at 5 µm and mounted on adhesive glass slides (Lecia Biosystems, UK). Slides were deparaffinised in xylene and rehydrated through a graded series of alcohols to water. Slides were washed with Tris-buffered saline and Tween 20 (TBST), and endogenous peroxidase activity was quenched with 3% hydrogen peroxide for 30 min. Antigen retrieval was performed on an 85 °C hot plate using citrate buffer for 10 min for sclerostin or 20 min in proteinase K (20 µg/ml) for DKK-1. Non-specific reactivity was blocked in TBST-5% bovine serum albumin (BSA) for 30 min at room temperature. Representative slides were incubated overnight at 4 °C with rabbit anti-human DKK-1 (Sigma-Aldrich), rabbit anti-human SOST (ABGENT, USA) antibodies (1:200 dilution).

After primary antibody incubation reaction, appropriate secondary biotinylated antibody (Vector Laboratories) was applied for 30 min at room temperature. Sections then were rinsed with TBST, and visualized using the avidin–biotin peroxidase diluted at 1:200 for 30 min. Sections were rinsed with TBST and treated with DAB (Vector labs, UK: 3, 3 diaminobenzidine). Sections were counterstained with haematoxylin or methyl green for 10 s, washed under running water for 30 s, dehydrated in ethanol, cleared in xylene, and cover slipped with DEPEX mounting medium. Sections were examined using an Olympus BX40 light microscope, and photographs were captured at 4–×20 magnifications. For general morphological analysis, decalcified sections were stained with Mayer’s H&E, and negative control sections were incubated with 1:200 dilution of the non-immunized rabbit serum (R&D Systems, UK).

### Statistical analysis

For quantification of histological staining, images of subchondral bone surfaces were captured at ×10 magnification from each slide, and cross-sectional areas of the subchondral bone plates were measured with ImageJ software (µm^2^). Total number of positive cells were counted manually under light microscope and plotted against subchondral bone thickness and represented as number of positive cells per µm^2^ as mean ± SEM from 6 slides per each core from 4 femoral head biopsies. Statistical analyses were carried out using SPSS version 11.0 for windows (SPSS Inc., Chicago, IL, USA). Subchondral bone thicknesses and number of positive cells per µm^2^ were compared by One-Way analysis of variance (ANOVA), using Tukey’s multiple comparison test, and a *P* value of <0.05 was regarded to indicate a significant difference.

## Results

### Immunohistochemistry

In an attempt to identify whether the presence or absence of cartilage, partial cartilage defect or presence of osteophytes would affect the thickness of the subchondral plate the structural parameters of subchondral bone plate were evaluated (Fig. 1a–e). There was no significant difference in subchondral bone thickness in cores taken from regions of macroscopically normal cartilage (100%) and partial thickness cartilage defect (103%) (Fig. 1e, core 1 and core 2). Subchondral bone plate was significantly thicker in cores taken from regions with full cartilage defect (207.2%) compared to cores with macroscopically normal cartilage, partial cartilage defect and subchondral bone of cores taken from osteophytes (*P* < 0.001, Fig. 1e). Subchondral bone plate was thinnest in cores taken from osteophytes (27.5%) (*P* < 0.05, Fig. 1e).

**Fig. 2:**
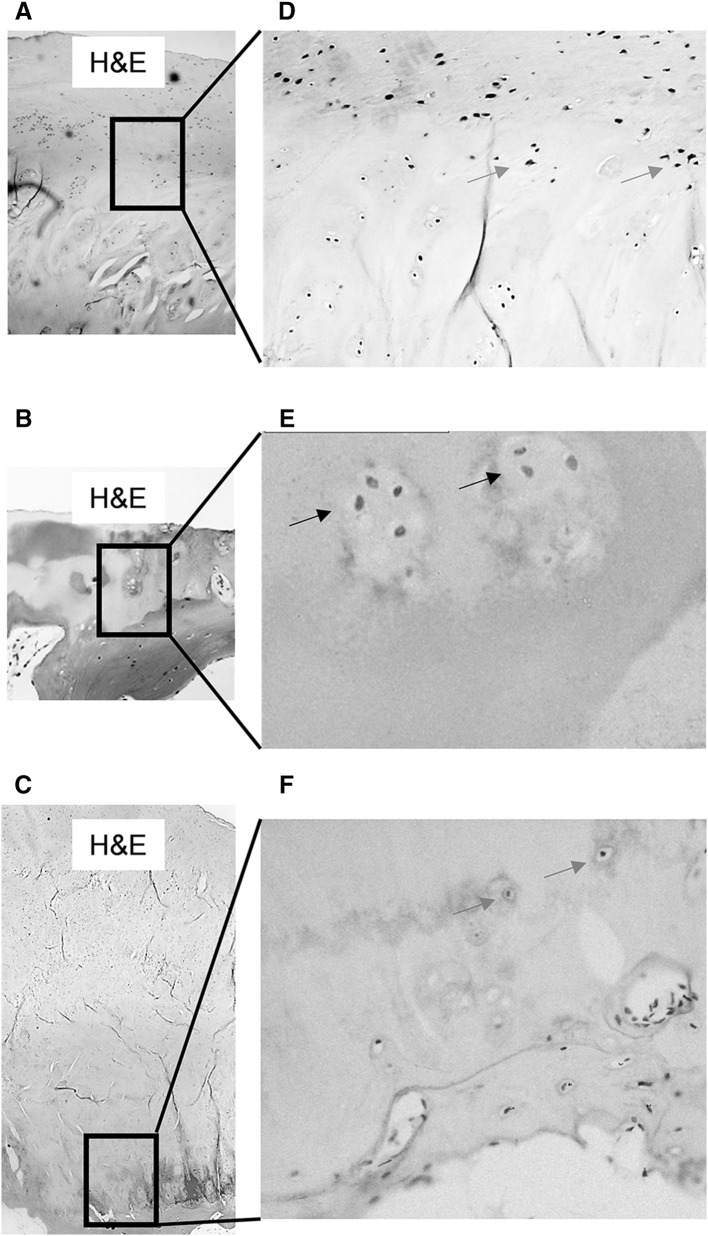
DKK-1 expression is high in OA chondrocytes Cylindrical cores were taken from OA femoral head biopsies, fixed in paraformaldehyde, decalcified in EDTA, 5 µm serial sections were cut longitudinally and stained with anti-human DKK-1 antibody. **a**–**c** Representative *H&E* staining of cartilage in cores taken from macroscopically normal cartilage, partial cartilage, and osteophyte regions. DKK-1 immunostaining only observed in chondrocytes in cores taken from macroscopically normal cartilage (*blue arrows* in **d**), but not in partial cartilage defect (*black arrows* in **e**) or osteophyte regions (*red arrows* in **f**) respectively. **a**–**c** ×4, **d** and **f** ×10 and **e** ×20 magnification

**Fig. 3:**
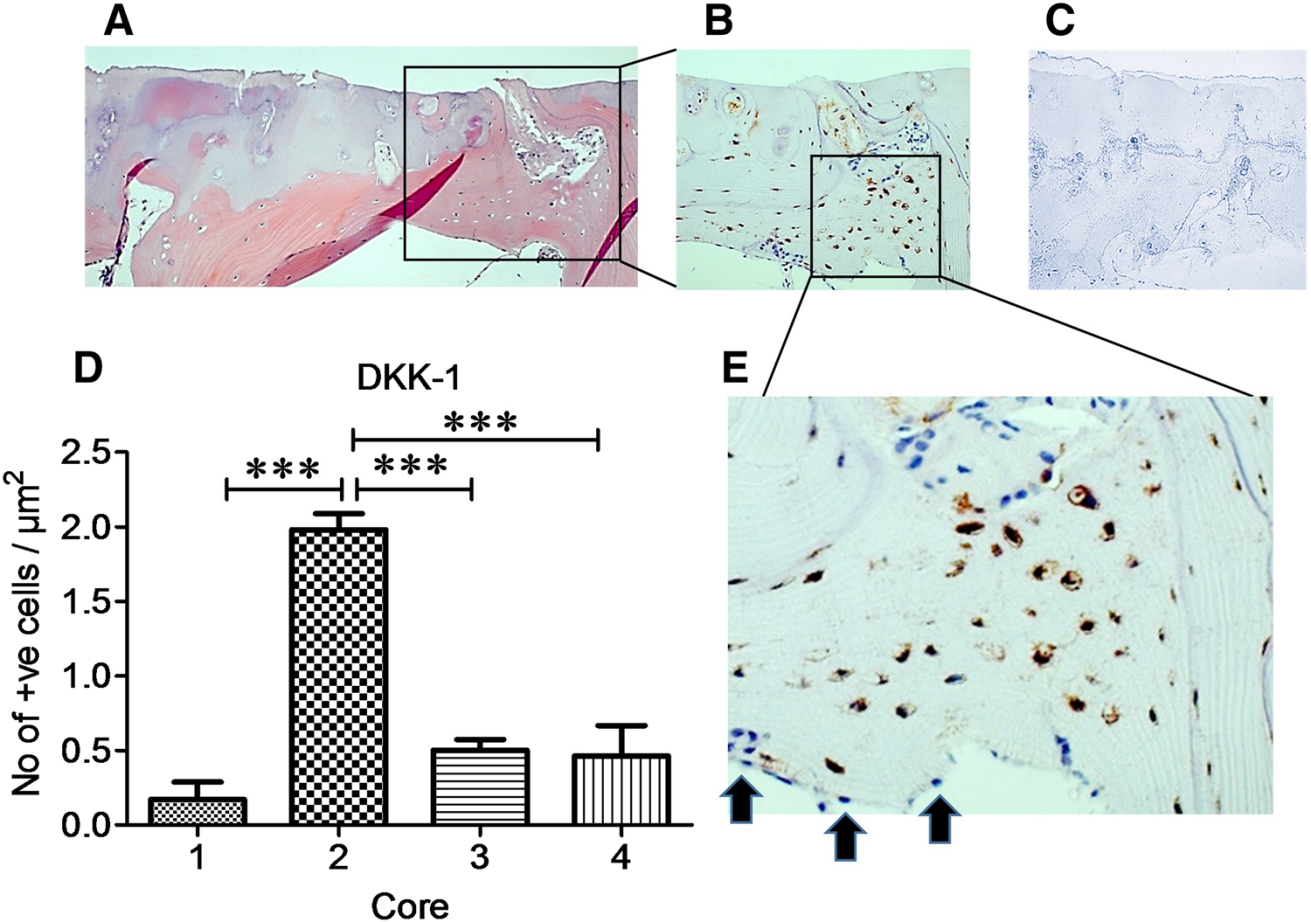
DKK-1 is over-expressed in osteocytes in subchondral bone underlying OA partial cartilage defect Cylindrical cores were taken from OA femoral head biopsies, fixed in paraformaldehyde, decalcified in EDTA, serial 5 µm sections were cut longitudinally and stained with anti-human DKK-1 antibody. **a**–**c** Representative staining of serial sections of subchondral bone of core taken from partial cartilage defect, **a** *H&E* staining, **b** DKK-1 immunostaining, **c** IgG negative staining, **d** quantification of DKK-1 immunoreactive osteocytes in subchondral bone from macroscopically normal cartilage cores (*1*), partial cartilage defect (*2*), full cartilage defect (*3*), and osteophyte (*4*). **e** High magnification of immunoreactive osteocytes from slide B. No immunoreactivity was observed in osteoblasts lining the subchondral bone plate (*black arrows* in **e**). **a**–**c** x10 and **e** x20 magnification. Data presented as mean ± SEM (One-way analysis of variance, Tukey’s multiple comparison test) of positive osteocytes per µm^2^ from 6 slides per each core from 4 femoral head biopsies compared to macroscopically normal cartilage. ***P < 0.001 for comparison of core 2 with other cores

**Fig. 4:**
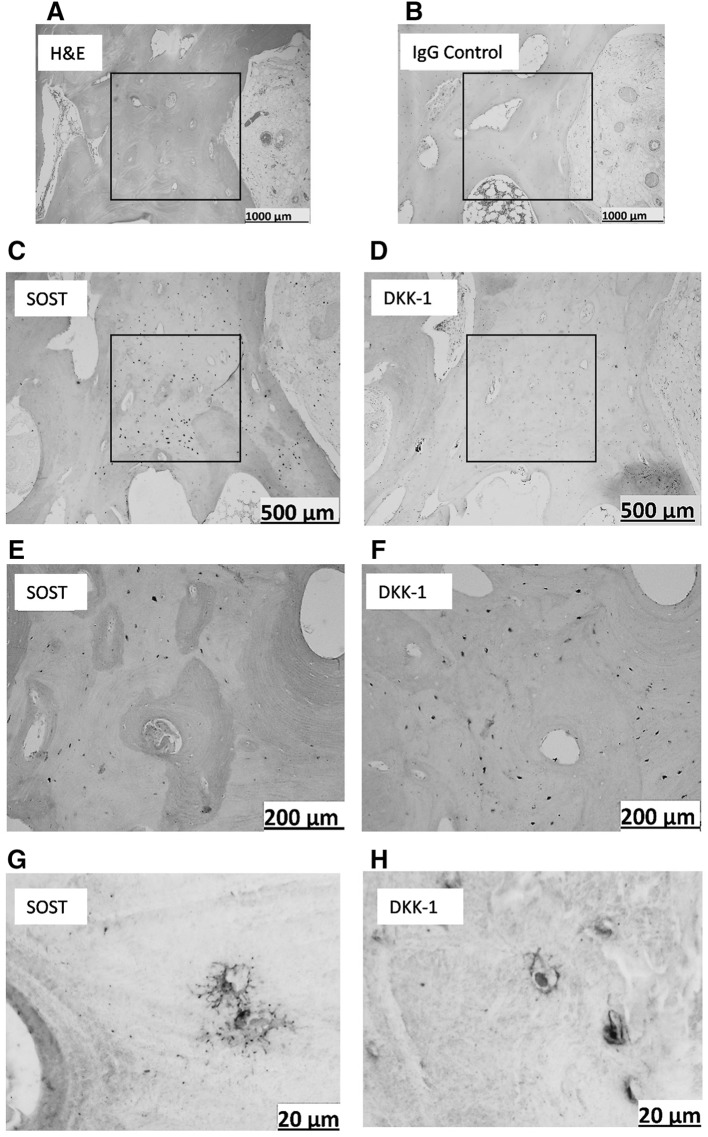
Co-expression of DKK-1 and SOST in OA trabecular bone. Representative staining of serial sections of trabecular bone in core taken from region of partial cartilage defect. **a** *H&E* staining, **b** IgG negative staining, **c, e, g** SOST immunostaining, **d, f, h** DKK-1 immunostaining. **a, b** ×4 and **c**–**f** ×10, **g**–**h** ×20 magnification

When OA femoral head cores were examined for DKK-1 immunoreactivity, surface chondrocytes in macroscopically normal cartilage regions (Fig. 2d, blue arrows) expressed abundant DKK-1. This pattern of DKK-1 expression was not observed in chondrocytes in cores taken from partial cartilage defect (Fig. 2e, black arrows) or in osteophyte cores (Fig. 2f), red arrows]. However, in bone tissue, staining was seen exclusively in osteocytes in subchondral bone plates (Fig. 3b, e). DKK-1 expression was significantly higher in subchondral bone of cores taken from regions with partial cartilage defects compared to subchondral bone in macroscopically normal cartilage, full cartilage defect, or osteophyte (*P* < 0.0001, Fig. 3d). It was of interest that osteocytes residing in trabecular bone in partial defect cores co-expressed both DKK-1 and SOST, the observation that was not evident in other cores (Fig. 4c–h).

**Fig. 5:**
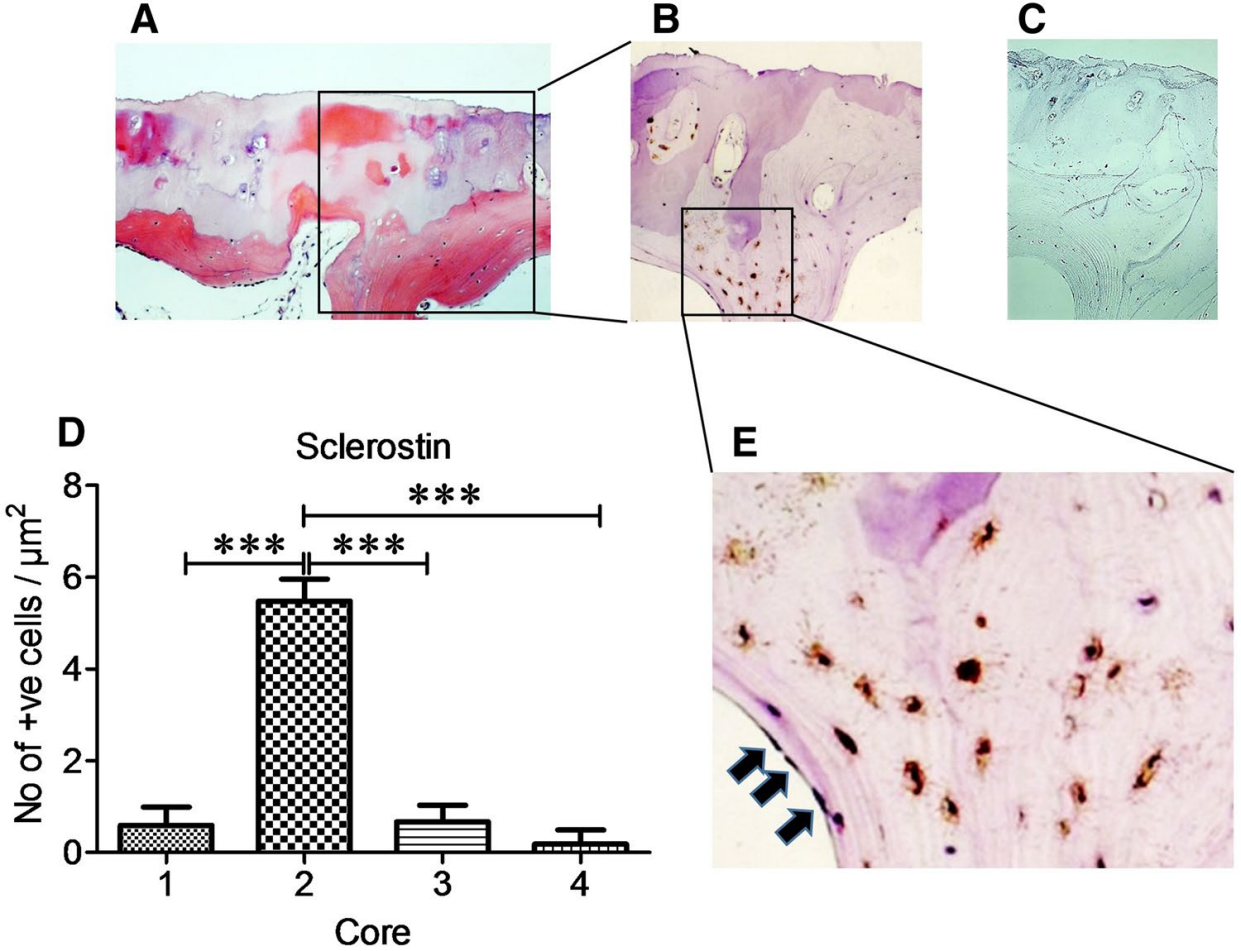
SOST is over-expressed in subchondral bone osteocytes underlying partial cartilage defect (**a**–**c**) Representative staining of serial sections of subchondral bone of cores taken from region with partial cartilage defect. **a** *H&E* staining, **b** SOST immunostaining, **c** IgG negative staining, **d** quantification of SOST immunoreactive osteocytes in subchondral bone from macroscopically normal cartilage (*core 1*), partial cartilage (*core 2*), full cartilage defect (*core 3*) and osteophyte (*core 4*). **e** High magnification of immunoreactive osteocytes from slide **b. a**–**c** ×10 and **e** ×20 magnification. Data presented as mean ± SEM (One-way analysis of variance, Tukey’s multiple comparison test) of positive osteocytes per µm^2^ from 6 slides per each core from 4 femoral head biopsies. ***P < 0.001 for comparison of immunoreactive osteocytes from cores taken from partial cartilage defect (*cores 2*) to other cores

**Fig. 6:**
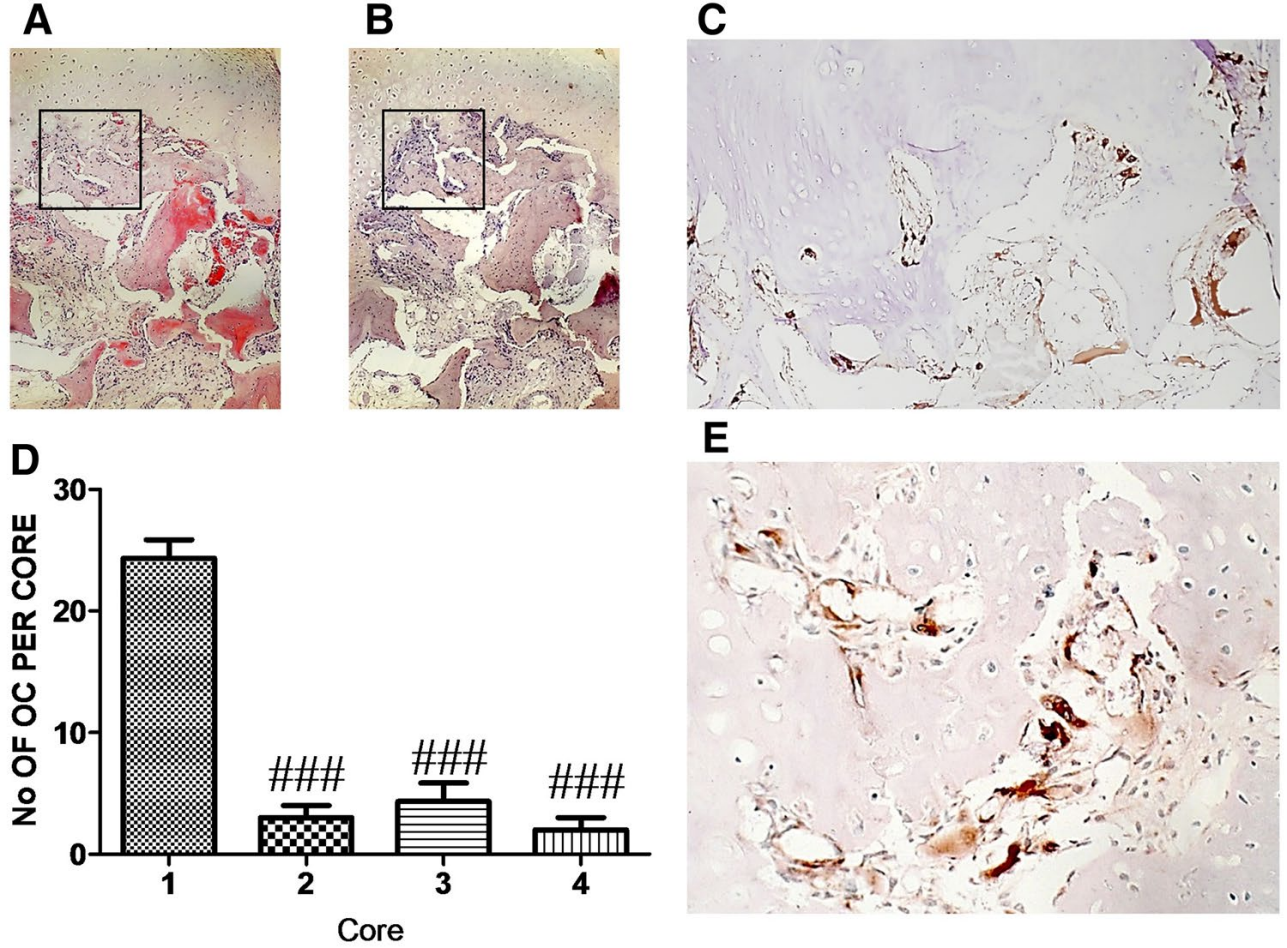
Osteoclast number is high in OA macroscopically normal cartilage Cylindrical cores were taken from OA femoral head biopsies, fixed in paraformaldehyde, decalcified in EDTA, 5 µm serial sections were cut longitudinally, and stained with anti-human CATK antibody. **a**–**c** Representative staining of serial sections of cartilage in core taken from full thickness cartilage. **a** *H&E* staining, **b** IgG negative staining, **c** CATK immunostaining, **d** quantification of CATK positive osteoclasts (OC) in cores from full thickness cartilage (*1*), partial cartilage (*2*), full cartilage defect (*3*) and osteophyte (*4*). **e** High magnification of immunoreactive osteoclasts from slide **c. a** and **b** ×4, **c** ×10 and **e** ×20 magnification. Data presented as mean ± SEM (One-way analysis of variance, Tukey’s multiple comparison test) of positive osteoclasts from 6 slides per each core from 4 femoral head biopsies. ### *P* < 0.001 for comparison of immunoreactive osteoclasts from cores relative to core 1

Similar to DKK-1 expression, SOST expression in OA cores was exclusively in osteocytes (Fig. 5b, e) and at highest levels in cores taken from partial cartilage defect compared to macroscopically normal cartilage, full cartilage defect, and osteophyte cores (*P* < 0.001, Fig. 5d). SOST was not expressed in chondrocytes and no DKK-1 or SOST immunoreactivity was observed in osteoblasts lining subchondral bone plate (Figs. 3e, 5e, black arrows). Osteoclasts were most abundant in the bone underlying macroscopically normal cartilage, as evidenced by Cathepsin K staining (Fig. 6).

## Discussion

This study has demonstrated that DKK-1 and SOST expression is highest in osteocytes underlying actively eroding cartilage (partial defects) and lowest under full thickness cartilage defects and macroscopically normal cartilage (Table 1). In direct contrast, subchondral bone is thickest underneath full thickness cartilage defects. Subchondral bone is known to be a very dynamic structure and adapted to mechanical forces that are frequently imposed across the joint [33]. Osteocytes are the only permanently resident cells within the subchondral bone, and our data further elaborate that alteration in the overlying cartilage thickness as a result of disease is associated with changes in Wnt activity.

**Table 1:** Immunohistochemical expression of DKK-1, SOST and osteoclast number in OA cores

	CORE 1	CORE 2	CORE 3	CORE 4
	Macroscopically normal cartilage	Partial cartilage defect	Full cartilage defect	Osteophyte
Cartilage depth	++++	++	**-**	n/a
Subchondral bone depth	++	++	++++	+
DKK-1	+	++++	++	++
SOST	+	++++	+	+
Osteoclast number	++++	+	+	+

Histological analysis of OA bone in this study has revealed that osteocyte cells exclusively co-express the Wnt inhibitors DKK-1 and SOST. It has been previously shown that chondrocytes in OA also express DKK-1 [23], and we also observed that this expression was only in the superficial layer of cartilage in our samples but not in mid or deep zones of cartilage. Furthermore, in our histological studies, DKK-1 was only seen in chondrocytes in cores taken from regions with macroscopically normal cartilage, and no expression was seen in other cores such as partial defect, or osteophytes. Additionally, histological analysis of serial sections also revealed that chondrocytes that expressed DKK-1 did not express SOST, and that no expression of SOST was observed in any chondrocytes in any of the cores.

The fact that subchondral bone was thicker in full cartilage defects coincides with the lack or lower expression of both DKK-1 and SOST and suggests higher Wnt activity. The higher expression of DKK-1 and SOST in osteocytes in cores taken from partial cartilage defect regions may reflect changes in loading as well as signaling from the adjacent eroding cartilage. Although we mainly focused on patterns of expression of these two markers in subchondral bone, it is interesting to note that osteocytes residing in trabecular bone in partial defect cores also predominantly co-expressed both DKK-1 and SOST. This expression was completely absent in trabecular bone of other cores.

Another finding of this study was the presence of giant multi-nucleated osteoclasts apparently resorbing cartilage in cores taken from macroscopically normal cartilage regions. This expression was only seen where subchondral bone had been invaded by bone marrow. It has been previously shown that osteoclasts are capable of resorbing calcified cartilage [34]; however, what signals these osteoclasts to appear in macroscopically normal cartilage remains unknown.

Given the cross-sectional nature of this method, we were unable to determine cause from effect of the observed findings and OA progression. As an experimental limitation, the analysis in this study represents a pilot work on relatively low sample number, and a larger cohort study could further confirm differential patterns of expression of Wnt antagonists in various regions of OA hip. In summary, we showed that subchondral bone thickness changes during the pathogenesis of OA by designing a method of taking cores from different regions of OA bone. Using this technique, we revealed that different compartments of bone within the same sample show different patterns of expression of Wnt antagonists. This system also shows that a dynamic sequence of changes are evident in osteocyte cells within subchondral bone. Whether these changes are caused by altered mechanical loading and have any consequences on cartilage degradation or bone remodeling is unclear.

Without direct measurements, it is not possible to confirm loading but hip lesions typically develop in the same position, such that Core 3 will have been subjected to the greatest cumulative loading, and therefore, the cartilage lesion develops here as well as the sclerotic bone. In contrast Core 1 is never directly loaded in the hip. Hip OA patients typically adjust their stance and gait to relieve painful pressure. Studies on osteophyte growth suggest that loading is likely to be highest in the proximity of an osteophyte, or Core 4. This may mean that Core 2 is newly unloaded or less loaded, consistent with the upregulation of Wnt antagonists, or that Core 2 is under altered load due to the altered matrix composition in active catalytic cartilage degradation, the combination of which drives upregulation of Wnt antagonists.

## Conclusions

This study is the first to demonstrate that osteocytes in subchondral bone co-express two inhibitors of the Wnt signaling pathway. The current study suggests a role for osteocytes in the structure and pathological remodeling of subchondral bone. It is of interest to find out what the effects of these inhibitors would be on other cells in OA bone such as osteoclasts. Further studies on the role of osteocytes in subchondral bone in OA may ultimately provide therapeutic targets for treatments of OA.
